# Structural analysis of new compound heterozygous variants in *PEPD* gene identified in a patient with Prolidase Deficiency diagnosed by exome sequencing

**DOI:** 10.1590/1678-4685-GMB-2020-0393

**Published:** 2021-04-19

**Authors:** Natália D. Linhares, Piotr Wilk, Elżbieta Wątor, Meire A. Tostes, Manfred S. Weiss, Sergio D. J. Pena

**Affiliations:** 1 Universidade Federal de Minas Gerais, Faculdade de Medicina, Laboratório de Genômica Clínica, Belo Horizonte, MG, Brazil.; 2 Universidade Federal de Minas Gerais, Instituto de Ciências Biológicas, Departamento de Bioquímica e Imunologia, Belo Horizonte, MG, Brazil.; 3 Helmholtz-Zentrum Berlin, Macromolecular Crystallography (HZB-MX), Berlin, Germany.; 4 Jagiellonian University, Malopolska Centre of Biotechnology, Kraków, Poland.; 5 Hospital das Clínicas da Universidade Federal de Minas Gerais, Serviço de Hematologia, Belo Horizonte, MG, Brazil.; 6 Laboratório Gene - Núcleo de Genética Médica, Belo Horizonte, MG, Brazil.

**Keywords:** Prolidase Deficiency, PEPD, exome sequencing, crystallization studies, metalloprotease

## Abstract

Prolidase Deficiency (PD) is an autosomal recessive rare disorder caused by loss or reduction of prolidase enzymatic activity due to variants in the *PEPD* gene. PD clinical features vary among affected individuals: skin ulcerations*,* recurrent infections*,* and developmental delay are common. In this study*,* we describe a 16-year-old boy with a mild PD phenotype comprising chronic eczema*,* recurrent infections and elevated IgE. Whole exome sequencing analysis revealed three *PEPD* variants: c.575T>C p.(Leu192Pro) inherited from the mother*,* and c.692_694del p.(Tyr231del) and c.1409G>A p.(Arg470His)*,* both inherited from the father. The variant p.(Tyr231del) has been previously characterized by high-resolution X-ray structure analysis as altering protein dynamics/flexibility. In order to study the effects of the other two prolidase variants*,* we performed site directed mutagenesis purification and crystallization studies. A high-resolution X-ray structure could only be obtained for the p.(Arg470His) variant*,* which showed no significant structural differences in comparison to WT prolidase. On the other hand*,* the p.(Leu192Pro) variant led to significant protein destabilization. Hence*,* we conclude that the maternal p.(Leu192Pro) variant was likely causally associated with the proband´s disease*,* together with the known pathogenic paternal variant p.(Tyr231del). Our results demonstrated the utility of exome sequencing to perform diagnosis in PD cases with mild phenotype.

## Introduction

Prolidase deficiency (PD) is a rare disorder caused by homozygous or compound heterozygous variants in the *PEPD* gene (Peptidase D*,* OMIM*613230). It is characterized by cutaneous lesions*,* recurrent infections principally of the skin and respiratory tract*,* dysmorphic facial features such as proptosis*,* hypertelorism*,* prominent forehead and micrognathia*,* hepatomegaly with elevated liver enzymes and splenomegaly (OMIM#170100). Anemia*,* thrombocytopenia*,* hypergammaglobulinemia and hypocomplementemia are common (Ferreira and Wang, 2015). Some patients may also have intellectual and developmental delays (reviewed in Lupi et al., 2008). The severity of symptoms in PD varies greatly among affected individuals; intrafamilial variability has been reported and some patients may apparently remain asymptomatic (Falik-Zaccai et al., 2010).

A recent review showed that 75 patients have been reported with a molecular diagnosis of PD (Spodenkiewicz et al., 2020); however*,* this number is certainly underestimated due to under-recognition of the disease by physicians. No curative treatment is available*,* although supportive treatment of skin*,* lung*,* and immunologic manifestations has been efficacious in some patients (Ferreira and Wang, 2015). Among the 75 PD patients reported*,* 35 variants were found in the *PEPD* gene*,* the majority of them is located in exons 8*,* 12 and 14 (Spodenkiewicz *et al.,* 2020). Until now*,* only 13 single nucleotide variants of *PEPD* have been classified as pathogenic or likely pathogenic in the ClinVar database (Landrum et al., 2018) - eight of these were missense variants. Using high-resolution X-ray crystal structure analysis of the PEPD protein prolidase*,* eight pathogenic variants have been characterized and possible mechanisms of prolidase enzymatic inactivation have been proposed (Wilk et al., 2017; Wilk *et al.,* 2018).

The *PEPD* gene encodes the enzyme peptidase D (EC 3.4.13.9)*,* also known as prolidase*,* which is the only enzyme in humans capable of hydrolysis of dipeptides containing proline (Pro) or hydroxyproline (Hyp) on their C-terminus. Consequently*,* patients with PD excrete very high amounts of imidodipeptides in the urine (Myara et al., 1984). Prolidase is particularly important in the degradation of collagens*,* a family of proteins where proline and hydroxyproline account for up to 22% of the amino acid content (Grant and Prockop, 1972). Collagens are the most abundant proteins in the human body and they exhibit an ubiquitous tissue distribution (Brinckmann, 2005).

Although there is considerable knowledge concerning the role of the prolidase enzyme (Myara et al., 1984; Wilk et al., 2020)*,* the pathophysiology of PD is not well understood (Lupi et al., 2008). Culture fibroblast from patients with PD showed necrosis-like cellular death and accumulation of the Gly-Pro dipeptides (Forlino et al., 2002). The necrosis seems to be related to the rupture of the cytoplasmatic membrane and the subsequent release of cell contents into the surrounding tissues. This*,* in turn*,* may cause an inflammatory response that could be responsible for the typical skin lesions in PD (Forlino *et al.,* 2002).

Ultrastructural studies of autopsy specimens of a PD case also showed morphological abnormalities seem to be one of causes of the clinical symptomatology*,* such as lamellar changes and splitting of the basement membrane of epidermal*,* dermal blood vessels*,* renal tubules*,* interstitial blood vessels and glomerular capillaries (Sekiya et al., 1985). Modifications of collagen structure could also contribute to the skin lesions; electron microscopic studies of PD patients´ apparently normal skin showed decreased size of collagen fibers and smaller and more heterogeneous fibril diameters (Leoni et al., 1987). Additionally*,* recent studies focusing on the cerebellar cortex showed a possible cellular/molecular basis of the intellectual disability in PD patients: mouse knocked-out for prolidase presented thinner collagen fibers and disorganized basement membrane below the pial meninx*,* which is required for correct cortical development (Insolia et al., 2020).

Here we describe a 16-year-old Brazilian boy with PD diagnosed through whole exome sequencing analysis. The patient presented three rare *PEPD* variants: c.575T>C p.(Leu192Pro) inherited from the mother*,* and c.692_694del p.(Tyr231del) and c.1409G>A p.(Arg470His) inherited from the father. One of these variants*,* the deletion of Tyr231 located at the dimer interface*,* has previously been characterized by us and classified as “structurally silent”*,* but exhibiting an alteration of the protein dynamics/flexibility (Wilk *et al.,* 2018). Here*,* we used high-resolution X-ray crystal structure analysis as our main tool to learn about the mechanism of inactivation of the other two prolidase variants. The observed implications of these single amino acid substitutions on the three-dimensional structure and activity of human prolidase are discussed.

## Subjects and Methods

### Case report

The proband was a 16-year-old Brazilian boy first seen with clinical picture of congenital immunodeficiency. He presented diffuse maculopapular eczema mainly in the flexural areas of the skin*,* ears*,* perineum and scalp noted since his first months of life*,* in addition to hepatosplenomegaly*,* facial dysmorphism (ocular proptosis*,* short nasal bridge*,* large and protruding ears)*,* recurrent infections (including skin infections caused by *S. aureus* and *Candida sp.,* pneumonia*,* sinusitis*,* otitis and urinary tract infection)*,* learning disabilities and cognitive delay. Blood count*,* myelogram and bone marrow karyotype were normal. At age 13*,* immunoglobulins IgA*,* IgG and IgM were normal and IgE was elevated (2508*,*2 UI/mL*,* normal values for age: 1*.*9 to 170*.*0 UI/mL). His parents were not consanguineous*,* and he had a healthy older sister and one younger brother*,* who had died at two months of age with a phenotype similar to that of the proband.

### Samples, DNA isolation and whole exome sequencing

The Research Ethics Committee of the Hospital das Clínicas of the Universidade Federal de Minas Gerais approved the study protocol. Informed consent was obtained according to current ethical and legal guidelines. The study was conducted in accordance with the Declaration of Helsinki. Genomic DNA was isolated from peripheral blood of the patient and his parents using a modified salting out procedure (Miller et al., 1988). Whole exome sequencing was performed on the proband’s sample by Centogene*,* Rostock*,* Germany*,* using Illumina’s Nextera Rapid Capture Exome Kit (Illumina*,* Inc.*,* San Diego*,* CA*,* USA)*,* which covers 214*,*405 exons with a total size of about 37 Mb. The generated library was sequenced using a HiSeq 2500 Sequencer (Illumina*,* San Diego*,* CA*,* USA). The average coverage was 70-100X*,* with circa 93% of the target bases being covered at least at 20X and with 84% being covered at least at 30X. All data were aligned to the GRCh37/hg19 reference genome build via Burrows-Wheeler Aligner (BWA) aligner. Variants were called and quality trimmed using Genome Analysis Toolkit (GATK). Variants were filtered for rare variants (allele frequency 0.01) utilizing databases such as 1000 Genomes Phase 3*,* NHLBI Exome Sequencing Project (ESP6500)*,* Single Nucleotide Polymorphism database (dbSNP141) and GnomAD database using the Mendel*,*MD software developed *in-house* (Cardenas et al., 2017) and the ENLIS Genome Research software (Enlis Genomics*,* Berkeley*,* CA*,* USA). Only variants with impact moderate or high according to SNPeff were considered (Cingolani et al., 2012). To analyze the impact of the candidate variants the software Alamut Visual version 2.11.0 (Interactive Biosoftware) was used*,* which showed the alignment of orthologous genes and included several protein-function prediction tools: SIFT*,* PolyPhen-2*,* MutationTaster and Align GVGD (Tavtigian et al., 2006; Adzhubei et al., 2010; Schwarz et al., 2010; Sim et al., 2012). CADD and REVEL scores were also evaluated (Ioannidis et al., 2016; Rentzsch et al., 2019). Since the proband was Brazilian*,* the allele frequency of the candidate variants were also investigated in two Brazilian databases: the Online Archive of Brazilian Mutations (ABraOM)*,* a repository containing genomic variants from 1*,*171 unrelated Brazilian individuals (Naslavsky et al., 2020); and the Brazilian Initiative on Precision Medicine Whole Exome Sequencing database (BIPMed-WES-db)*,* a database containing information obtained from 106 Brazilian subjects.

### Sanger sequencing

Sanger sequencing was performed in order to validate the variants of interest identified by exome analysis using the BigDye Terminator v3.1 Cycle Sequencing Kit (Applied Biosystems) and the Applied Biosystems (ABI) 3130 Genetic Analyzer. Sequencing data was analyzed using the software Sequencher version 4.1.4 (Gene Codes Corporation).

### Hydroxyproline quantification

In order to confirm the diagnosis of PD*,* total hydroxyproline was quantified in the patient´s urine sample by high performance liquid chromatography. Before quantification*,* the urine samples were exposed to acid hydrolysis (using 6 N HCl)*,* which gives rise to a marked increase in hydroxyproline (Ferreira and Wang, 2015; Ferreira and Cusmano-Ozog*,* 2017).

### Site-directed mutagenesis

The p.(Arg470His) (hProlR470H) and p.(Leu192Pro) (hProlL192P) point mutations were introduced in a pET-28a vector containing the WT *PEPD* gene using the QuickChange Site-Directed Mutagenesis Kit (Agilent Technologies) following the manufacturer’s manual and verified by DNA sequencing.

### Protein production and purification of hProlR470H

hProlR470H was expressed and purified as previously described for other *PEPD* variants (Wilk *et al.,* 2018). In short*, E. coli* BL21 (DE3) Rosetta2 cells were transformed with the pET-28a vector containing the sequence encoding the PEPD variant p.(Arg470His). Cells were cultured in Terrific Broth media supplemented with necessary antibiotics. Protein expression was induced by the addition of 1 mM IPTG and conducted overnight at 18°C. Upon cell disruption the protein was purified by affinity chromatography using a 5 mL HisTrap HP (GE Healthcare Europe GmbH*,* Freiburg*,* Germany) chromatography column. The protein was subjected to TEV protease cleavage in order to remove the HisTag and subsequently to size-exclusion chromatography (SEC) on a HiLoad 16/60 Superdex 200 column. The purity of the final preparation was evaluated by SDS-PAGE. Fractions of highest purity were pooled*,* concentrated to 25 mg/ml*,* aliquoted and flash frozen in liquid nitrogen.

### Protein production and purification of hProlL192P

Preliminary expression tests using different *E. coli* strains (BL21 (DE3)*,* Arctic Express*,* Rosetta2) transformed with the pET-28a vector containing gene with the desired variant showed no soluble expression. Therefore*,* for further optimization of expression two fusion protein constructs were used: pMCSG9 (HisTag-MBP-TEV cleavage site - PEPD) and pCryst+ (HisTag-SUMO-T4 Lysozyme - PEPD). These constructs were subsequently used for transformation of *E. coli* Arctic Express cells*,* in which protein expression was induced with 0.5 mM IPTG and conducted at 5 °C. The general protein purification protocol was as described for the hProlR470H variant*,* yet the protein yield was approx. 10x lower (i.e. ca. 3 mg per 1 liter).

### Crystallization

Crystals of hProlR470H were obtained by sitting drop vapor diffusion technique at 293 K. Concentrated to 18-23 mg/ml*,* the protein was mixed with mother liquor consisting of 690-760 mM NaCitrate pH 7.4-8.2 and 10 mM NaTetraBorate (Na_2_B_4_O_7_). The obtained crystals were transferred to cryo-protectant solution containing 25% (v/v) glycerol in mother liquor supplemented with the ligands of interest (20 mM MnCl_2_ and 20 mM GlyPro). After soaking for 5 min*,* the crystals were flash-cooled in LN_2_.

For crystallization of the hProlL192P variant commercial random screens were used. Screens were set using protein expressed in both vectors (pMCS9*,* pCryst+). Crystals were obtained in several different conditions. Interestingly*,* in the same screening conditions (JCSG Core II: 0.2 M Magnesium chloride*,* 0.1 M Imidazole pH = 8.0*,* 40% MPD) crystals were obtained from both expression conditions albeit with different morphologies.

### Diffraction data collection and structure determination

Crystals of two complexes of hProlR470H were measured at beamlines BL14.1 and BL14.2 at the BESSY II electron storage ring (HZB*,* Berlin*,* Germany) (Mueller et al., 2015). For each selected crystal*,* two diffraction data sets*,* a native 0.9184 Å and a long-wavelength 1.8871 Å (corresponding to Mn X-ray absorption edge) were acquired. The diffraction data were processed using XDSAPP (Sparta et al., 2016). The structure of the hProlR470H variant was solved with Phaser (McCoy et al., 2007) using human wild-type prolidase (PDB-Id: 5M4G) as a search model (Wilk et al., 2017). The obtained models were further rebuilt and refined using COOT (Emsley et al., 2010) and Phenix.refine (Afonine et al., 2012). The quality of the model was validated using the MolProbity server (Chen et al., 2010). Anomalous difference electron density maps were calculated using ANODE (Thorn and Sheldrick, 2011) from the long-wavelength data sets for the identification of the manganese ions. The final coordinates and structure factors for the substrate-free and the substrate bound structure have been deposited in the Protein Data Bank (PDB*,* 2019) with accession number 6QSB and 6QSC*,* respectively. The corresponding raw diffraction images were uploaded to the Integrated Resource for Reproducibility in Macromolecular Crystallography (IRRMC) (Grabowski et al., 2016). Relevant data collection*,* processing and refinement statistics were presented in Table 1.

**Table 1:** - Data collection, processing and refinement statistics for hProlR470H variant. The numbers in parentheses refer to the highest resolution shell of the data.

	p.(Arg470His) variant with Mn ions (6QSB)		p.(Arg470His) variant with Mn ions and GlyPro ligand (6QSC)	
Wavelength (Å)	0.9184	1.8912	0.9184	1.8912
Resolution range	23.4 - 1.99 (2.06 - 1.99)	48.3-2.55 (2.70-2.55)	48.35 - 1.569 (1.625 - 1.569)	48.38-1.83 (1.94-1.83)
Space group	C222_1_	C222_1_	C222_1_	C222_1_
Unit cell (Å)	103.56 108.53 211.80, 90 90 90	103.64 108.53 211.82, 90 90 90	103.54 108.71 211.55, 90 90 90	103.62 108.80 211.71, 90 90 90
Total reflections	561664 (91853)	507743 (72464)	1239456 (202299)	1054776 (44031)
Unique reflections	81732 (7983)	75340 (11971)	165436 (26391)	187658 (19205)
Multiplicity	6.9	6.7	7.5	5.6
Completeness (%)	99.6 (98.7)	99.5 (97.8)	99.8 (98.9)	91.8 (58.2)
Mean I/sigma(I)	5.4 (0.6)	7.9 (1.8)	16.6 (1.5)	18.2 (1.0)
Wilson B-factor (Å^2^)	35.4	36.0	22.8	38.8
R-merge (%)	34.2 (284.2)	20.8 (91.4)	7.1 (121.2)	5.1 (67.7)
R-meas (%)	36.8 (306.7)	22.6 (100.0)	7.7 (129.9)	5.5 (84.9)
CC_1/2_	98.8 (23.5)	98.9 (67.6)	99.9 (64.9)	99.9 (58.4)
Reflections used in refinement	81662 (7978)		165419 (16219)	
Reflections used for R-free	2097 (205)		2100 (206)	
R-work (%)	18.83 (35.23)		14.68 (30.20)	
R-free (%)	22.33 (41.05)		16.74 (34.61)	
Number of non-hydrogen atoms	8349		8865	
Macromolecules	7524		7675	
Ligands	46		60	
Solvent	779		1130	
Protein residues	962		964	
RMS(bonds)	0.005		0.009	
RMS(angles)	0.69		0.90	
Ramachandran favored (%)	98.33		98.01	
Ramachandran allowed (%)	1.67		1.99	
Ramachandran outliers (%)	0.00		0.00	
Rotamer outliers (%)	0.12		0.24	
Clashscore	3.14		3.45	
Average B-factor (Å^2^)	39.37		30.08	
Macromolecules	39.04		28.65	
Ligands	59.96		55.52	
Solvent	41.31		38.38	

In case of the hProlL192P variant*,* the obtained crystals were tested for diffraction. The obtained diffraction limit was approx. 8-12Å*,* which was not sufficient for structure determination. So far*,* the crystals could not be improved after optimization.

### Structure analysis

For proper placement of the structure within the unit cell the ACHESYM server was used (Kowiel *et al.,* 2014). In order to assess the effect of the variants on the prolidase structure*,* the p.(Arg470His) variant structure was compared to the previously characterized structure of wild type human Prolidase (hProl). Normalized B-factors were calculated by dividing individual B-factor value by the average B-factor of a given structure. The average was taken from all protein atoms. For visualization the PyMOL software was used.

## Results

### Molecular analysis

**Figure 1 -:**
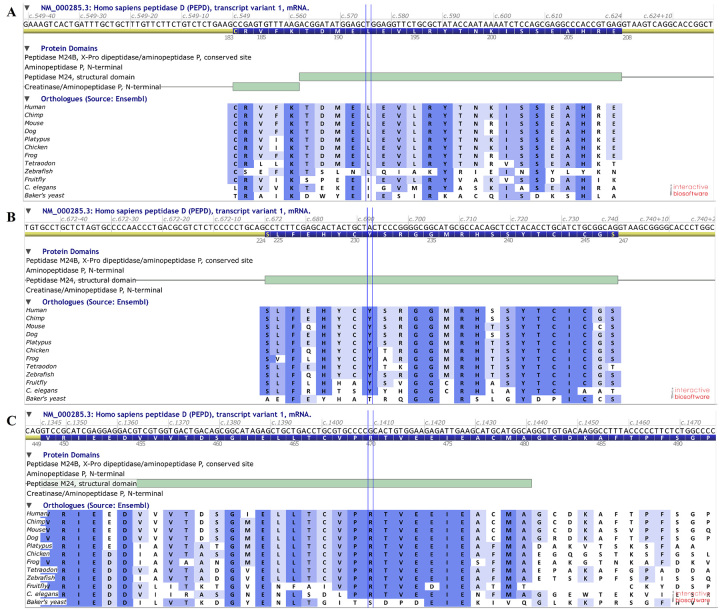
Comparative analysis using PEPD orthologs. Part of PEPD protein showing variant c.575T>C p.(Leu192Pro) (A), c.692_694del p.(Tyr231del) (B) and c.1409G>A p.(Arg470His) (C). The positions of the variants were indicated by vertical blue lines. Fully conserved amino acids are marked in dark blue and less-conserved amino acids are in lighter blue colors. All variants are located in regions well conserved throughout evolution. Alignments of PEPD orthologues were made with software Alamut Visual version 2.11.0.

Exome sequencing analysis resulted in the identification of three rare heterozygous missense variants in*PEPD* (NM_000285.3). The first variant was located in exon 8 position Chr19:33954942A>G (hg19) c.575T>C p.(Leu192Pro)*,* it has not been previously described either in patients with PD or in healthy individuals from worldwide populations*,* according to the gnomAD database (Karczewski et al., 2019) and ABraOM (Naslavsky et al., 2020). It had *in silico* pathogenic characteristics as assessed by the prediction programs SIFT (deleterious; score = 0.01)*,* PolyPhen-2 (probably damaging; score = 0.988)*,* MutationTaster (disease causing; p-value = 1)*,* Align GVGD (Class C65)*,* CADD (score 27)*,* REVEL (score 0.907). The second variant was a deletion of three bases that led to the loss of residue Tyr231 in position c.692_694del p.(Tyr231del) in exon 10 (Chr19:33904527_33904529del); it was previously registered in the dbSNP dataset under the number rs745834191 and has been catalogued as pathogenic/likely pathogenic in the ClinVar database (Accession VCV000328810) (Landrum et al., 2018). Its allelic frequency was 0.08% in ABraOM*,* and 0.01% in worldwide populations according to the gnomAD database; 39 heterozygous individuals were reported and 11 of them were from Latino population and 27 were European (non-Finnish). The third variant was located in exon 15 position Chr19:33878323C>T c.1409G>A p.(Arg470His)*,* it has not been previously described in patients with PD. It was registered in the dbSNP database under the number rs765552774*,* and its allelic frequency was 0.08% in ABraOM and 0.01% in gnomAD database; 49 heterozygous individuals were reported and 44 were from Latino population. It had*in silico* pathogenic characteristics as assessed by the prediction programs SIFT (deleterious; score = 0.02)*,* PolyPhen-2 (probably damaging; score = 1.00)*,* MutationTaster (disease causing; p-value = 1)*,* Align GVGD (class C0)*,* CADD (score 32)*,* REVEL (score 0.749). All three variants affected evolutionarily conserved amino acids*,* which was evidence of biological importance (Figure 1). None of the three variants were found in BIPMed-WES-db.

**Figure 2 -:**
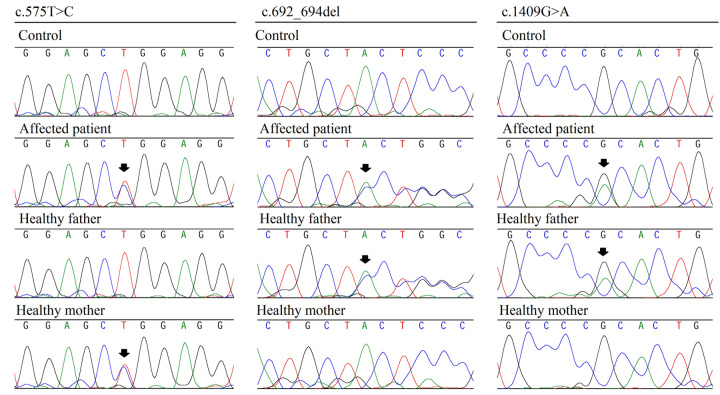
Sanger sequencing results of *PEPD* gene. Black arrows indicate the position of the variants. Variant c.575T>C p.(Leu192Pro) was inherited from the mother*,* and variants c.692_694del p.(Tyr231del) and c.1409G>A p.(Arg470His) were inherited from the father.

These three *PEPD* variants were validated by Sanger sequencing in samples from the parents*,* proband and control (Figure 2). Molecular studies of the parents showed that variant c.575T>C p.(Leu192Pro) was inherited from the mother*,* while the variants c.692_694del p.(Tyr231del) and c.1409G>A p.(Arg470His) were both inherited from the father.

### Prolidase biochemical confirmation

In order to confirm the diagnosis of Prolidase deficiency*,* total hydroxyproline was quantified in the patient´s urine sample and showed high levels of excretion: 241 mg / 24 h (normal values: 16 to 40 mg / 24h).

### Overall structure of p.(Arg470His) variant of human prolidase

**Figure 3 -:**
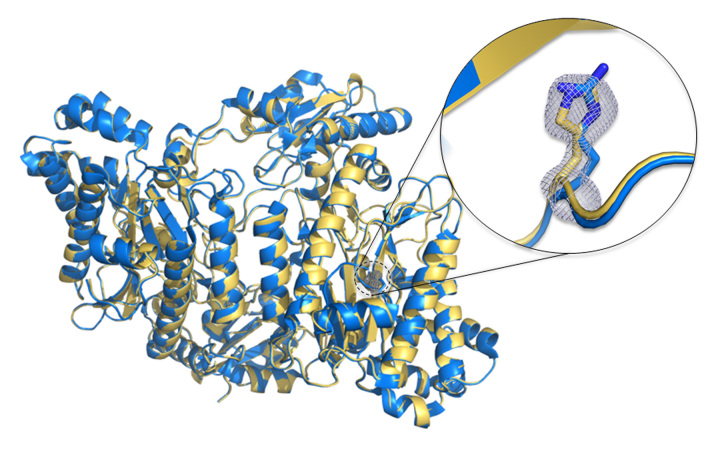
Comparison of WT prolidase (in blue) and the p.(Arg470His) variant (in yellow). The inlet shows magnification of described modification. The His470 side chain is presented with a difference electron density omit map contoured at 3σ.

We presented here two high-resolution crystal structures for the p.(Arg470His) variant of human prolidase. The first structure (deposited in the PDB with the accession Id 6QSB) was the substrate-free structure*,* which represented the active enzyme state before the binding of the substrate. In the second structure (deposited in the PDB with the accession Id 6QSC) the GlyPro substrate was present in both active sites of the dimer*,* which was content of the asymmetric unit of the crystal. The electron density maps clearly indicated the presence of an imidazol side chain rather than a guanidinum group at amino acid position 470*,* proving the success of the introduced amino acid substitution (see Figure 3). All relevant statistics concerning diffraction data collection*,* structure determination and validation were given in Table 1. The final model of the p.(Arg470His) variant with Mn^2+^ ions (PDB-Id: 6QSB) comprised residues 6-484 in chain A and 6-488 in chain B. In both active sites two manganese ions were present*,* with occupancies >0.95. The final model of the p.(Arg470His) variant with Mn ions and GlyPro ligand (PDB-Id: 6QSC) comprised residues 6-482 in chain A and 6-488 in chain B. In both monomers two manganese ions (one with hydroxide ion coordinated) and Gly-Pro ligand per active site were observed. Despite an apparently unaltered active site architecture*,* the bound substrate remains intact. This was in contrast to wild-type prolidase*,* where a substitution of Mn^2+^ by Mg^2+^ ions was necessary to capture the substrate-bound state. MolProbity analysis shows that all residues in both models were in the allowed regions of Ramachandran plot.

### The p.(Leu192Pro) variant of human prolidase

**Figure 4 -:**
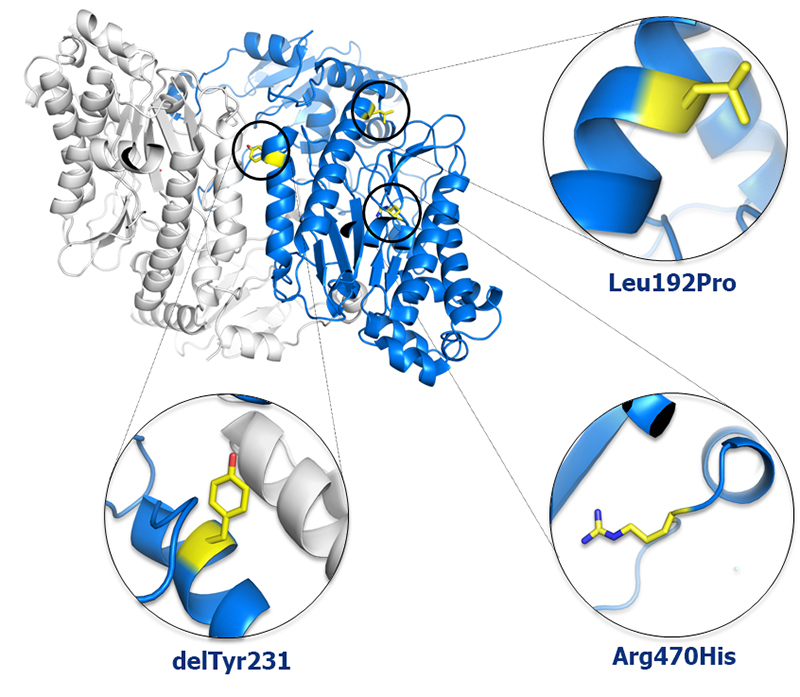
Location of discussed mutations in the hProl structure. Prolidase is shown in cartoon representation and two monomers are colored white and blue. The residues undergoing substitution are shown on one monomer as sticks with carbon atoms colored yellow.

The second variant of human prolidase discussed in this study was the p.(Leu192Pro) variant. Here*,* the amino acid Leu192 was substituted by the imino acid proline. Since the substitution site was located in the middle of an α-helix*,* its effect on the three-dimensional structure must be significant (see Figure 4). Compared to all other amino acids*,* proline is relatively rigid amino acid*,* which occupies a rather small area in the Ramachandran plot*,* due to its side chain ring structure. It has therefore a rather low α-helix forming propensity. Consequently*,* it is likely that the substitution of Leu for Pro leads to a breaking of the α-helix*,* and to a serious destabilization and/or misfolding of the variant protein. This notion was corroborated by our finding that most of the variant protein*,* which was overexpressed in bacterial cells*,* ended up in inclusion bodies*,* which is often the case for incorrectly folded proteins. Irrespective of that*,* a small amount of the variant protein could be obtained in a soluble form*,* which was then used for crystallization experiments. However*,* even after optimization*,* the obtained crystals were not of high enough quality for structure determination. In conclusion*,* we speculate that the p.(Leu192Pro) variant has a serious effect on the three-dimensional structure of the protein*,* leading to either a disruption of the active site of the enzyme or a complete misfolding of most of the expressed protein. This could explain the reduced prolidase activity*,* which was observed in the patient.

## Discussion

Here we present a patient with PD and new compound heterozygous variants: c.575T>C p.(Leu192Pro) was inherited from the mother*,* and c.692_694del p.(Tyr231del) and c.1409G>A p.(Arg470His) were inherited from the father. Variants p.(Leu192Pro) and p.(Arg470His) have never been described in patients with PD. The same 3-bp deletion p.(Tyr231del) was described in homozygous state in three unrelated Portuguese patients with prolidase deficiency (Lupi et al., 2004; Cottin et al., 2020). Lupi *et al.* (2004) showed that the expression level of the p.(Tyr231del) mutant transcript was similar to the control*,* however*,* the activity of the mutant enzyme was reduced to 5% of the control value in cultured skin fibroblasts. Moreover*,* further studies performed by our group demonstrated that the variant p.(Tyr231del) resulted in an increased active site flexibility and reduced thermal stability (Wilk *et al.,* 2018). We showed that previously reported pathogenic variants resulted in structures that may be divided into four groups depending on the presumed effect of the corresponding variants on the reaction mechanism: disruption of the catalytic Mn_2_(OH-)-center; introduction of chain disorder along with the displacement of important active site residues; rigidification of the active site; and flexibilization of the active site (Wilk *et al.,* 2018).

**Figure 5 -:**
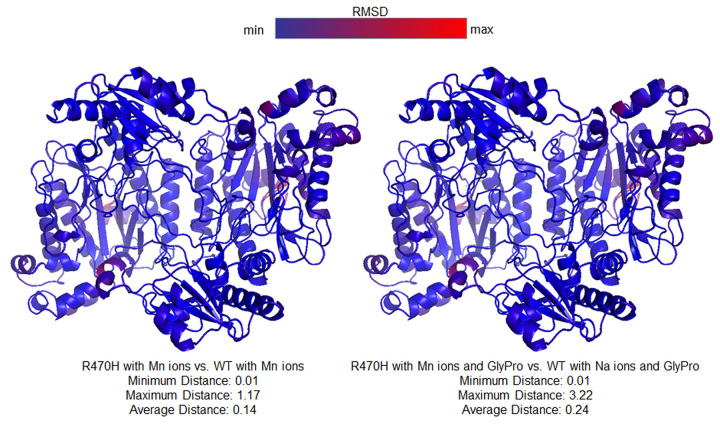
Comparison of the structures of the p.(Arg470His) variant and WT prolidase using Cα Root Mean Square Deviation. (A) structures with Mn ions only*,* (B) structures with Mn ions and GlyPro substrate.

Therefore*,* we expected that p.(Leu192Pro)*,* located *in trans* with the pathogenic p.(Tyr231del) in our patient*,* may have a damaging effect in the protein structure and that p.(Arg470His) may be benign. Accordingly*,* we showed here that p.(Leu192Pro) led to significant destabilization of the protein. In addition*,* comparison of structure of hProlArg470His to previously reported structure of hProl using Root Mean Square Deviation of Cα positions approach shows rather small structural differences mainly concentrated around poorly ordered C-termini (Figure 5). The only noticeable difference was the mutated residue.

**Figure 6 -:**
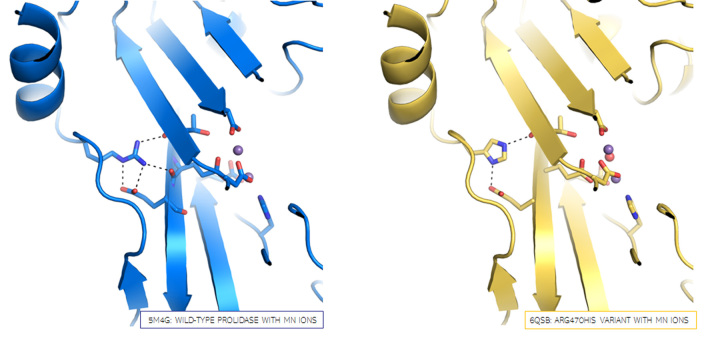
Comparison of bonding pattern of residue 470 in WT prolidase (in blue) and p.(Arg470His) variant (in yellow). Main chain is shown as cartoon representation. Mutated residue and residues forming metal binding cleft of the active site are shown as sticks. Electrostatic interactions are shown as black dashed lines. Manganese ions are shown as magenta spheres.

In hProl*,* Arg470 was located close to the active site and it formed hydrogen bond/ salt bridges with 3 residues: Thr289*,* Glu452 and Glu453. Glu452 is part of the active site and it participates in catalysis by coordinating both manganese ions. In case of hProlArg470His*,* substitution of Arg by His resulted in lesser number of salt bridges. His470 forms interacted only with Thr289 and Glu453 (Figure 6).

**Figure 7 -:**
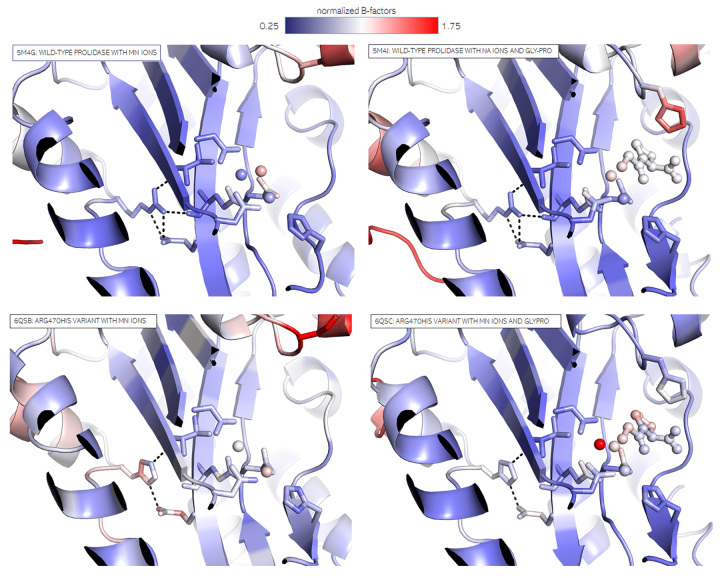
B-factor analysis of WT prolidase and the p.(Arg470His) variant with Mn ions only and in complex with substrate. Main chain is shown with cartoon representation. Mutated residue and residues forming active site are shown as sticks. Electrostatic interactions are shown as black dashed lines. The substrate is shown as ball-and-stick and ions as spheres. A slight red shift i.e. an increase of B-factor values in vicinity of the point of substitution is discernible.

In order to prepare more comprehensive analysis*,* we normalized B-factors in both structures of p.(Arg470His) variant and appropriate WT structures. Overall comparison of B-factors again showed rather no obvious differences. However*,* subtle changes could indeed be observed in active site. When comparing hProlR470H structures to hProl slightly higher B-factors of the His atoms than for the Arg atoms can be observed. This effect is more pronounced in the substrate-free structure. Significantly higher B-factors could be observed also for the GlyPro ligand and one of the Mn^2+^ ions in the structure of the pathological variant in complex with Mn ions and the GlyPro substrate. It seems that the lack of interaction with the catalytically important Mn-coordinating residue Glu452 could therefore cause flexibilization of the active site. This flexibilization also manifested itself as increased B-factor value of the Glu452 atoms (Figure 7).

Altogether*,* we speculated that the Arg470 played a role in stabilizing the architecture of the active site and that small changes in this region could interfere with the catalytic efficiency. However*,* in light of our results*,* the protein with the variant p.(Arg470His) seems to be stable and unlikely to trigger cell response due to presence of misfolded protein. In addition*,* previous studies suggest that as little as 10% of prolidase activity can significantly ameliorate clinical manifestation (Besio *et al.,* 2013; Wator et al., 2020). Taking into account potential residual activity of Arg470His*,* this variant can be considered benign. On the contrary*,* the p.Leu192Pro variant had a dramatic effect on the protein and is likely to cause aggregates of misfolded protein in the cell and is unlikely to retain any catalytic activity.

The *in silico* prediction programs (such as SIFT*,* PolyPhen-2*,* MutationTaster*,* CADD and REVEL) assessed the variant p.(Arg470His) as having pathogenic characteristics. Though*,* by high-resolution X-ray diffraction analysis*,* the variant p.(Arg470His) could be classified as “structurally silent”*,* because there is the lack of observable significant differences in the 3D-structure of the protein*,* understood as relative position of all amino acids (expressed as per atom coordinates). This difference could be explained because *in silico* programs are solely based on sequence homology considerations as well as on the physico-chemical similarity between the alternate amino acids. Considering our experimental results*,* we have applied the ACMG guidelines and reclassified the p.(Arg470His) variant to likely benign (Richards et al., 2015). Consequently*,* we believe that p.(Arg470His) is not needed to cause the patient’s disease*,* although it could be influencing it.

PD is a disease known to have highly variable expression with incomplete penetrance (Lupi et al., 2006). For example*,* two sisters described with the same variant p.(Glu412Lys) differed significantly in phenotype: one had the typical PD symptoms with 21 years old*,* while the other sister was found to be asymptomatic at age 29 (Lupi *et al.,* 2006). Clinical manifestations of PD frequently are detectable in infancy or early childhood - for instance skin ulcers can begin as early as age six months (Mandel et al., 2000). Yet*,* a case of clinical onset at age 30 years has been described (Dyne et al., 2001).

In addition to variable expressivity*,* another characteristic of PD that complicates its diagnosis is that it could be misdiagnosed as an immunodeficiency*,* which was the case of the patient described here. This occurred because patients with PD have overlapping phenotypic features with hyper IgE syndrome such as severe eczema*,* and recurrent skin and lung infections (OMIM#243700). Notably*,* our patient presented a mild phenotype as he did not present the typically severe*,* chronic*,* recalcitrant*,* and painful skin ulcers of the lower extremities*,* seen in 61% of PD patients (Ferreira and Wang, 2015; Spodenkiewicz et al., 2020)*,* although the amount of secreted urinary dipeptides confirmed his molecular diagnosis. The elevated IgE*,* chronic eczema*,* recurrent infections*,* splenomegaly and hepatomegaly gave a clue towards the diagnosis*,* since they have been described in 64%*,* 58%*,* 76%*,* 72% and 53% of PD patients*,* respectively (Spodenkiewicz *et al.,* 2020).

## Conclusions

The case presented here demonstrates the utility of whole exome sequencing to perform diagnosis in a PD case with mild phenotype*,* first misdiagnosed as congenital immunodeficiency. We provided here important genetic and structural characterization of two new variants present in the proband*,* contributing to our understanding of PD pathophysiology*,* which has disorders with Hyper IgE as differential diagnosis. Based on our analysis we can classify the hProlArg470His variant of human prolidase as “structurally silent” and assume that loss of function of this particular mutant can arise from flexibilization of the active site. The nature of the second variant p.(Leu192Pro) is likely to lead to significant destabilization of the whole protein. For further investigation of the nature of both mutants*,* activity and stability tests are essential. The obtained structural information about *PEPD* variants can significantly advance our understanding of PD pathology*,* which is still not fully understood.

## References

[B1] Adzhubei IA, Schmidt S, Peshkin L, Ramensky VE, Gerasimova A, Bork P, Kondrashov AS, Sunyaev SR (2010). A method and server for predicting damaging missense
mutations. Nat Methods.

[B2] Afonine PV, Grosse-Kunstleve RW, Echols N, Headd JJ, Moriarty NW, Mustyakimov M, Terwilliger TC, Urzhumtsev A, Zwart PH, Adams PD (2012). Towards automated crystallographic structure refinement with
phenix.refine. Acta Crystallogr D Biol Crystallogr.

[B3] Besio R, Gioia R, Cossu F, Monzani E, Nicolis S, Cucca L, Profumo A, Casella L, Tenni R, Bolognesi M (2013). Kinetic and structural evidences on human prolidase pathological
mutants suggest strategies for enzyme functional rescue. PLoS One.

[B4] Brinckmann J (2005). Collagens at a Glance. Top Curr Chem.

[B5] Cardenas RGCCL, Linhares ND, Ferreira RL, Pena SDJ (2017). Mendel,MD: A user-friendly open-source web tool for analyzing WES
and WGS in the diagnosis of patients with Mendelian
disorders. PLoS Comput Biol.

[B6] Chen VB, Arendall WB, Headd JJ, Keedy DA, Immormino RM, Kapral GJ, Murray LW, Richardson JS, Richardson DC (2010). MolProbity: all-atom structure validation for macromolecular
crystallography. Acta Crystallogr D Biol Crystallogr.

[B7] Cingolani P, Platts A, Wang Le Lily, Coon M, Nguyen T, Wang L, Land SJ, Lu X, Ruden DM (2012). A program for annotating and predicting the effects of single
nucleotide polymorphisms, SnpEff: SNPs in the genome of Drosophila
melanogaster strain w1118; iso-2; iso-3. Fly.

[B8] Cottin V, Nasser M, Traclet J, Chalabreysse L, Lebre AS, Si-Mohamed S, Philit F, Thivolet-Bejui F (2020). Prolidase deficiency: a new genetic cause of combined pulmonary
fibrosis and emphysema syndrome in the adult. Eur Respir J.

[B9] Dyne K, Zanaboni G, Bertazzoni M, Cetta G, Viglio S, Lupi A, Iadarola P (2001). Mild, late-onset prolidase deficiency: another Italian
case. Br J Dermatol.

[B10] Emsley P, Lohkamp B, Scott WG, Cowtan K (2010). Features and development of Coot. Acta Crystallogr D Biol Crystallogr.

[B11] Falik-Zaccai TC, Khayat M, Luder A, Frenkel P, Magen D, Brik R, Gershoni-Baruch R, Mandel H (2010). A broad spectrum of developmental delay in a large cohort of
prolidase deficiency patients demonstrates marked interfamilial and
intrafamilial phenotypic variability. Am J Med Genet B Neuropsychiatr Genet.

[B12] Ferreira C, Wang H, Pagon RA, Bird TD, Dolan CR, k Stephens, Adam MP (2015). Prolidase Deficiency. GeneReviews™.

[B13] Ferreira CR, Cusmano-Ozog K (2017). Spurious elevation of multiple urine amino acids by Ion-Exchange
Chromatography in patients with Prolidase Deficiency. JIMD Rep.

[B14] Forlino A, Lupi A, Vaghi P, Cornaglia AI, Calligaro A, Campari E, Cetta G (2002). Mutation analysis of five new patients affected by prolidase
deficiency: the lack of enzyme activity causes necrosis-like cell death in
cultured fibroblasts. Hum Genet.

[B15] Grabowski M, Langner KM, Cymborowski M, Porebski PJ, Sroka P, Zheng H, Cooper DR, Zimmerman MD, Elsliger M-A, Burley SK (2016). A public database of macromolecular diffraction
experiments. Acta Crystallogr D Struct Biol.

[B16] Grant ME, Prockop DJ (1972). The biosynthesis of collagen. 1. N Engl J Med.

[B17] Insolia V, Priori EC, Gasperini C, Coppa F, Cocchia M, Iervasi E, Ferrari B, Besio R, Maruelli S, Bernocchi G (2020). Prolidase enzyme is required for extracellular matrix integrity
and impacts on postnatal cerebellar cortex development. J Comp Neurol.

[B18] Ioannidis NM, Rothstein JH, Pejaver V, Middha S, McDonnell SK, Baheti S, Musolf A, Li Q, Holzinger E, Karyadi D (2016). REVEL: An ensemble method for predicting the pathogenicity of
rare missense variants. Am J Hum Genet.

[B19] Karczewski KJ, Francioli LC, Tiao G, Cummings BB, Alföldi J, Wang Q, Collins RL, Laricchia KM, Ganna A, Birnbaum DP (2019). Variation across 141,456 human exomes and genomes reveals the
spectrum of loss-of-function intolerance across human protein-coding
genes. bioRxiv.

[B20] Kowiel M, Jaskolski M, Dauter Z (2014). ACHESYM: an algorithm and server for standardized placement of
macromolecular models in the unit cell. Acta Crystallogr D Biol Crystallogr.

[B21] Landrum MJ, Lee JM, Benson M, Brown GR, Chao C, Chitipiralla S, Gu B, Hart J, Hoffman D, Jang W (2018). ClinVar: improving access to variant interpretations and
supporting evidence. Nucleic Acids Res.

[B22] Leoni A, Cetta G, Tenni R, Pasquali-Ronchetti I, Bertolini F, Guerra D, Dyne K, Castellani A (1987). Prolidase deficiency in two siblings with chronic leg
ulcerations. Clinical, biochemical, and morphologic aspects. Arch Dermatol.

[B23] Lupi A, De Riso A, Della Torre S, Rossi A, Campari E, Vilarinho L, Cetta G, Forlino A (2004). Characterization of a new PEPD allele causing prolidase
deficiency in two unrelated patients: natural-occurrent mutations as a tool
to investigate structure-function relationship. J Hum Genet.

[B24] Lupi A, Rossi A, Campari E, Pecora F, Lund AM, Elcioglu NH, Gultepe M, Di Rocco M, Cetta G, Forlino A (2006). Molecular characterisation of six patients with prolidase
deficiency: identification of the first small duplication in the prolidase
gene and of a mutation generating symptomatic and asymptomatic outcomes
within the same family. J Med Genet.

[B25] Lupi A, Tenni R, Rossi A, Cetta G, Forlino A (2008). Human prolidase and prolidase deficiency: an overview on the
characterization of the enzyme involved in proline recycling and on the
effects of its mutations. Amino Acids.

[B26] Mandel H, Abeling N, Gutman A, Berant M, Scholten EG, Sheiman C, Luder A, van Gennip AH (2000). Prolidase deficiency among an Israeli population: prenatal
diagnosis in a genetic disorder with uncertain prognosis. Prenat Diagn.

[B27] McCoy A, Grosse-Kunstleve R, Adams P, Winn M, Storoni L (2007). PHASER crystallographic software. J Appl Cryst.

[B28] Miller SA, Dykes DD, Polesky HF (1988). A simple salting out procedure for extracting DNA from human
nucleated cells. Nucleic Acids Res.

[B29] Mueller U, Förster R, Hellmig M, Huschmann FU, Kastner A, Malecki P, Pühringer S, Röwer M, Sparta K, Steffien M (2015). The macromolecular crystallography beamlines at BESSY II of the
Helmholtz-Zentrum Berlin: Current status and perspectives. Eur Phys J Plus.

[B30] Myara I, Charpentier C, Lemonnier A (1984). Prolidase and prolidase deficiency. Life Sci.

[B31] Naslavsky MS, Scliar MO, Yamamoto GL, Wang JYT, Zverinova S, Karp T, Nunes K, Ceroni JRM, de Carvalho DL, da Silva Simões CE (2020). Whole-genome sequencing of 1,171 elderly admixed individuals from
the largest Latin American metropolis (São Paulo, Brazil). bioRxiv.

[B32] wwPDB consortium (2019). Protein Data Bank: the single global archive for 3D
macromolecular structure data. Nucleic Acids Res.

[B33] Rentzsch P, Witten D, Cooper GM, Shendure J, Kircher M (2019). CADD: predicting the deleteriousness of variants throughout the
human genome. Nucleic Acids Res.

[B34] Richards S, Aziz N, Bale S, Bick D, Das S, Gastier-Foster J, Grody WW, Hegde M, Lyon E, Spector E (2015). Standards and guidelines for the interpretation of sequence
variants: a joint consensus recommendation of the American College of
Medical Genetics and Genomics and the Association for Molecular
Pathology. Genet Med.

[B35] Schwarz JM, Rodelsperger C, Schuelke M, Seelow D (2010). MutationTaster evaluates disease-causing potential of sequence
alterations. Nat Methods.

[B36] Sekiya M, Ohnishi Y, Kimura K (1985). An autopsy case of prolidase deficiency. Virchows Arch A Pathol Anat Histopathol.

[B37] Sim NL, Kumar P, Hu J, Henikoff S, Schneider G, Ng PC (2012). SIFT web server: predicting effects of amino acid substitutions
on proteins. Nucleic Acids Res.

[B38] Sparta KM, Krug M, Heinemann U, Mueller U, Weiss MS (2016). XDSAPP2.0. J Appl Cryst.

[B39] Spodenkiewicz M, Spodenkiewicz M, Cleary M, Massier M, Fitsialos G, Cottin V, Jouret G, Poirsier C, Doco-Fenzy M, Lebre AS (2020). Clinical genetics of prolidase deficiency: An updated
review. Biology.

[B40] Tavtigian SV, Deffenbaugh AM, Yin L, Judkins T, Scholl T, Samollow PB, de Silva D, Zharkikh A, Thomas A (2006). Comprehensive statistical study of 452 BRCA1 missense
substitutions with classification of eight recurrent substitutions as
neutral. J Med Genet.

[B41] Thorn A, Sheldrick GM (2011). ANODE: anomalous and heavy-atom density
calculation. J Appl Cryst.

[B42] Wator E, Rutkiewicz M, Weiss MS, Wilk P (2020). Co-expression with chaperones can affect protein 3D structure as
exemplified by loss-of-function variants of human prolidase. FEBS Lett.

[B43] Wilk P, Uehlein M, Kalms J, Dobbek H, Mueller U, Weiss MS (2017). Substrate specificity and reaction mechanism of human
prolidase. FEBS J.

[B44] Wilk P, Uehlein M, Piwowarczyk R, Dobbek H, Mueller U, Weiss MS (2018). Structural basis for prolidase deficiency disease
mechanisms. FEBS J.

[B45] Wilk P, Wator E, Weiss MS (2020). Prolidase - A protein with many faces. Biochimie.

